# CDK4/6 inhibition: the late harvest cycle begins

**DOI:** 10.18632/oncotarget.9576

**Published:** 2016-05-24

**Authors:** Shom Goel, Jean J. Zhao

**Affiliations:** Department of Medical Oncology, Dana- Farber Cancer Institute, Boston MA, USA; Department of Cancer Biology, Dana- Farber Cancer Institute, Boston MA, USA; Department of Biological Chemistry and Molecular Pharmacology, Harvard Medical School, Boston MA, USA; The Broad Institute of MIT and Harvard, Cambridge, MA, USA

**Keywords:** CDK4/6, cyclin, cell cycle, cancer, abemaciclib

Effective and safe pharmacologic inhibition of the cyclin dependent kinases (CDKs) has been a goal of cancer researchers for many years. CDKs are attractive therapeutic targets in cancer for two main reasons - first, some tumors harbor dependencies on particular CDKs for their initiation and maintained growth; second, there is a surprising amount of redundancy between various CDKs in maintaining normal organ function [1, 2]. These observations suggest that it might be possible to inhibit a certain CDK to slow tumor growth without invoking prohibitive toxicity. Unfortunately, clinical trials using CDK inhibitors have largely failed due to low potency and specificity of the agents used.

Recently, however, medicinal chemists have developed a new generation of more potent and selective CDK inhibitors. Of these, inhibitors of CDK4 and CDK6 (“CDK4/6 inhibitors”) are the best characterized and have progressed furthest in clinical development. Indeed, recent trials have shown that the addition of palbociclib (a potent and selective CDK4/CDK6 inhibitor) to endocrine therapy significantly improves progression-free survival in women with advanced hormone receptor-positive breast cancer, leading to its accelerated approval by the FDA in 2015 [3]. This success has sparked enthusiasm for CDK4/6 inhibitors and a large number of trials testing CDK4/6 inhibition for a variety of tumor types have opened. Agents in development include palbociclib, abemaciclib, and ribociclib.

The canonical function of CDKs 4 and 6 is to facilitate cellular transition from G1 to S phase of the cell cycle. This is achieved when levels of a D-type cyclin (cyclin D1, D2, or D3) rise in G1, facilitating formation of a cyclin D-CDK4/6 holoenzyme (also incorporating a Cip/Kip subunit such as p21 or p27) [4]. The active kinase phosphorylates the retinoblastoma tumor suppressor protein (RB), liberating RB from E2F family transcription factors. This promotes expression of genes regulating the G1-S transition. It is not surprising, therefore, that pharmacologic CDK4/6 inhibitors suppress RB phosphorylation, induce G1 arrest, and ultimately produce a senescent-like phenotype in sensitive tumor cells. Indeed, these effects alone are likely to render these agents as effective therapies for many tumors.

Understanding these canonical CDK4/6 pathways might allow us to rapidly identify tumors that are likely to be sensitive to pharmacologic inhibition of these kinases. Potential candidates include: (i) tumors driven by oncogenes that are known to increase cyclin D protein levels (e.g. those that increase cyclin D transcription through the Ras-Raf-Mek-Erk pathway or cyclin D stability through the PI3K-AKT pathway) [5]; and (ii) those which might show increased dependency on CDK4/6 due to cyclin D gene amplification, loss of p16INK4a, or unique chromosomal rearrangements increasing cyclin D expression. Such tumors represent the low-hanging fruit - the soonest to be subjected to trials of CDK4/6 inhibition.

**Figure 1:**
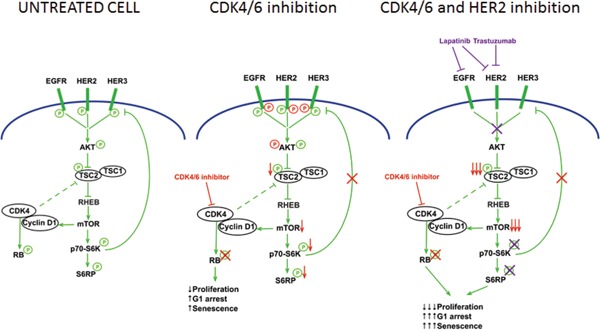
Exploiting a non-canonical CDK4/6 pathway to overcome drug resistance A non-canonical function of CDK4/6 that can be exploited for therapeutic benefit. Left: In a drug-resistant HER2-positive breast cancer cell, CDK4 phosphorylates RB and also interacts with TSC2. CDK4 activity enhances mTOR activity, leading to feedback suppression of upstream receptor tyrosine kinases (RTKs). Middle: When the cell is treated with a CDK4/6 inhibitor (red arrows), RB phosphorylation declines, suppressing cell proliferation. TSC2 phosphorylation also declines, reducing mTOR activity and relieving feedback inhibition on RTKs. Right: A CDK4/6 inhibitor- treated cell is thus primed to the effect of anti-HER2 therapy (e.g. lapatinib, trastuzumab). Combination therapy leads to more potent shutdown of mTOR activity and the combined mTOR/RB suppression enhances therapeutic effect. (Modified from Goel et al, Cancer Cell 2016 [6])

We would argue that there are also likely to be contexts in which CDK4/6 inhibitors show activity which are not so intuitive. The preclinical literature is replete with descriptions of non-canonical, kinase-dependent functions of CDKs 4 and 6 which CDK4/6 inhibitors would be expected to modulate. It is thus entirely conceivable that the cell cycle inhibitory properties of these agents might be accompanied by a host of other “on-target” effects.

As one example of this, we recently demonstrated a non-canonical pathway through which CDK4/6 inhibitors can be utilized to overcome resistance to other targeted therapies [6]. Our research has focused on resistance to anti-HER2 targeted therapies in breast cancers with amplification of the *ERBB2* oncogene (which encodes for the HER2 receptor tyrosine kinase). Our *in vitro*, *in vivo*, and molecular studies utilized models of therapy resistant HER2-positive breast cancer. In each case, we found that CDK4/6 inhibition with abemaciclib not only reduced RB phosphorylation as expected, but also reduced the phosphorylation of the tumor suppressor TSC2 (tuberin), leading to a partial suppression of downstream mTOR activity. Previous work has shown that in HER2-amplified breast cancers, a suppression of mTOR activity relieves feedback inhibition on upstream receptor tyrosine kinases, and indeed we observed increased phosphorylation of several EGFR-family kinases (EGFR, HER2, HER3) in the resistant tumors. This re-sensitizes cells to the effects of anti-HER2 therapy, and the net result is a potent synergistic interaction between HER2 and CDK4/6 inhibitors. As such, CDK4/6 inhibition overcomes resistance to anti-HER2 therapy.

Our findings are novel and represent a new potential use for CDK4/6 inhibitors in cancer. They have also led to the development of clinical trials testing the combination of HER2 and CDK4/6 blockade in patients with treatment- refractory HER2-positive breast cancer. Importantly, it is notable that Zacharek et al first presented the notion of TSC2 regulation by D-type cyclins and CDK4/6 in back in 2005 [7]. Using molecular biology techniques, they showed that the activity of CDK4/6 holoenzymes can, through TSC2 regulation, stimulate mTOR activity. Our finding that CDK4/6 inhibitors interfere with this process is therefore consistent with a previous description of a non-canonical function of CDK4/6. With the advent of CDK4/6 inhibitors, we now have a way to leverage this knowledge into a therapeutic benefit.

Indeed, we believe that there is much to be gained by performing a careful review of the CDK4/6 literature from decades past. Over this time, researchers have painstakingly characterized the functions of these kinases in models ranging from yeast to transgenic mice. We should use this information to ensure that we do not simply conceive of CDK4/6 inhibitors as cell cycle inhibitors, but rather as drugs that may have myriad other effects in tumor cells. It is time for a renaissance where we reap the rewards of the efforts of the pioneers in the field.

On this note, we suggest a number of CDK4/6- related topics that must be “re-examined” using the available pharmacological inhibitors. These include (i) interactions between CDK4/6 and other signaling pathways to optimize rational combination regimens for cancer (ii) the tumor cell senescence response to CDK4/6 inhibition must be put into context with the vast existing knowledge on tumor cell senescence; (iii) the effects of CDK6 on bone marrow function and hence the potential consequences of CDK4/6 inhibitors on the host immune environment; (iv) the specific differences between CDK4 and CDK6, especially considering that the available compounds inhibit these kinases in different proportions.

It is a rare situation in which newly developed kinase inhibitors can stand on the shoulders of over 30 years of intensive research into the very pathway they target. It is now incumbent on us to use our existing knowledge of CDKs 4 and 6 to rapidly optimize the use of CDK4/6 inhibitors. We may not need to re-invent the wheel, but rather use the agents as powerful tools to confirm previous findings, uncover new ones, and rapidly determine their relevance to cancer. Our patients have waited long enough.
